# Improving medical outcomes in lupus: enhancing the effectiveness of the medical interview and improving patient support

**DOI:** 10.1093/rap/rkaa066

**Published:** 2020-11-30

**Authors:** Sara Booth

**Affiliations:** 1 c/o Cambridge Breathlessness Intervention Service; 2 Palliative and Supportive Care, Cambridge University Hospitals NHS Foundation Trust, Cambridge, UK

**This editorial refers to ‘Is it me? The impact of patient—physician interactions on lupus patients’ psychological wellbeing, cognitions and healthcareseeking behaviour’, by Melanie Sloan et al. doi.org/10.1093/rap/rkaa037**

It is long established in reviews outlining best practice in managing SLE that ‘substantial issues remain in improving quality of life’ [1] and that ‘the morbidity of SLE remains considerable’. [2]. The focus of such articles remains finding ‘targeted therapies’ [2] to induce remission with minimal damage from ‘medication side effects’. [2] It is clear that this remains some way off, and yet strategies that might help patients currently living with lupus towards a better quality of life are not discussed with the same authority and energy or are not mentioned at all.

Using patient experiences, in a study built on a foundation of user participation from its outset in coalition with eminent rheumatologists, Sloan *et al.* [3] highlight the therapeutic importance of excellent medical communication, stressing its potential to improve medical outcomes and increase patients’ quality of life without morbidity and at low cost, with the added benefit of reducing time wasted in futile medical consultations.

Sloan *et al.* [3] briefly review the considerable evidence that people with lupus/UCTD often experience a harrowing diagnostic journey and live with a range of constitutional symptoms (most commonly, fatigue) that often remain unaddressed or inadequately managed. They have high levels of depression and anxiety, associated variously with the fluctuating and uncertain nature of SLE/UCTD and the psychosocial consequences of the illness, and yet few are offered psychological treatment. Difficulties in maintaining education or employment and claiming benefits are well documented, further damaging individuals’ physical and mental health and exacerbating poverty [4], and having the most profound impact on the young. The gap between patients’ and physicians’ concerns is highlighted again.

Using this evidence, Sloan *et al.* [3] accessed patient expertise via the LUPUS UK website, in order to understand more about patients’ current experiences of medical support in managing their illness. They surveyed 233 people, conducting qualitative interviews with 21 purposively sampled individuals, who provided evidence of the sorts of medical contacts that both help and hinder clinical improvement (Fig. 1). Reassuringly, the great majority reported at least one sustaining medical relationship with a positive impact on their psychological and physical health. Less happily, another major theme was ‘persisting insecurity and distrust…’ [3] from what were perceived as ‘…dismissive physician responses to symptoms’ [3], which had negative effects on individuals’ health behaviours, sometimes ‘for years’. [3] This was particularly important for those with UCTD or those diagnosed in childhood/teenage years, sometimes fraying family relationships when parents were felt to ‘side’ with doctors sceptical of patients’ symptoms.

**Fig. 1:**
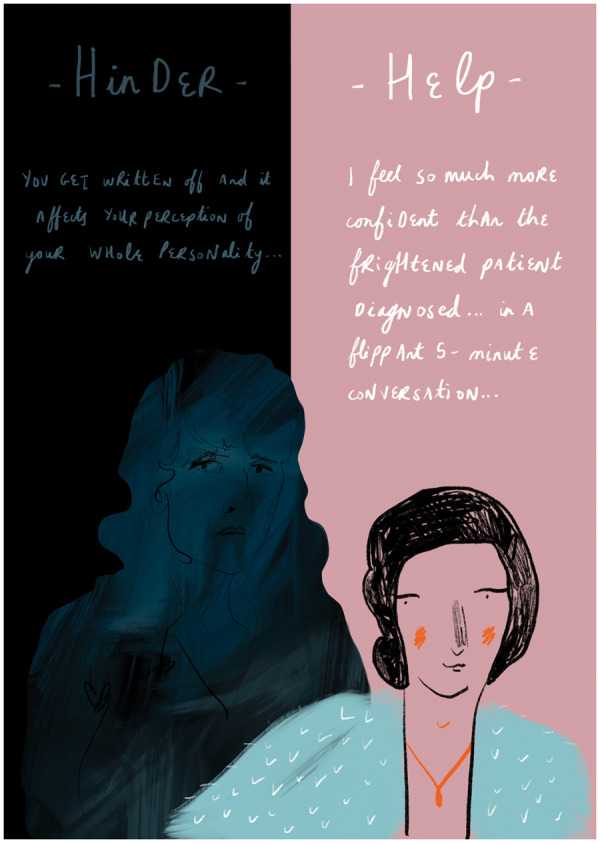
Words that hurt and words that heal

Many patients felt disbelieved when they described fatigue. This is not confined to lupus care [5]. The fatigue associated with illness is not tiredness, a mistake comparable to associating the exhilarating breathlessness a fit person feels after running with the terrifying dyspnoea that accompanies advanced respiratory disease [5] and leads to inappropriate management. Fatigue ‘incorporates total body feelings, ranging from tiredness to exhaustion creating an overall body condition which is unrelenting … alters the person’s ability to function’ [6]. In both symptoms, objective measurements are lacking; pulmonary function tests are a poor guide to the severity of dyspnoea. Fatigue contributes to loss of employment [4] and reduces quality of life and cardiovascular fitness. The importance of fatigue [7] has long been recognized, but many doctors feel ill equipped to make management recommendations when it is raised. The science of symptom control is expanding, although most is based in palliative and supportive care. A psychophysical rehabilitative approach, shaped by individual assessment, is needed [8]. Exercise is helpful and health building; however, simply advising ‘exercise’ to someone who volunteers feeling utterly exhausted is likely (as Sloan’s findings confirm) to make them feel they have not been heard, initiating a downward cycle of trust and inhibiting future patient communication.

The authors acknowledge the limitation of their sample. Most are highly educated, Caucasian women >40 years of age. Those most disadvantaged by SLE are those already disadvantaged by racism, poverty and lack of educational opportunities. They are either unemployed, on benefits, or in low-paid manual, insecure work, powerless to modify its content or conditions. Their voices are rarely heard in lupus research, and we need to capture their needs and aspirations in order to improve care and advocate effectively. It is essential that future researchers find ways to document and develop interventions to change the experiences of the most disadvantaged people with lupus. Medical advocacy is necessary, stressing that financial barriers to health care and the limitations of medicine in the presence of wide social inequality prevent effective disease management, before the disadvantaged will have the best health possible.

Another concern is the authors repeating, without comment, the term ‘post-traumatic stress disorder’ (PTSD) [3], where it has been used by participants. This a narrowly defined, contested psychiatric diagnosis, and what is described by Sloan *et al.* would not fit the Diagnostic Statistical Manual (DSM) criteria [9], whereby an experience of an imminent threat to life or physical integrity is needed to make the diagnosis. It is clear that some medical encounters have left deep psychological scars that impair future medical communication. There are continuing discussions about ‘multiple psychological micro-traumas’ enabling a diagnosis of PTSD to be made, but these are still theoretical [10]. It might be more helpful to continue to describe a taxonomy of *sustaining* (in order that they can be replicated) or *depleting* (in order that they can be avoided) consultation styles, the latter leading to poorer medical outcomes, and to produce practical guidance for training in communication skills in specialist lupus units. Given that lupus/UCTD patients already face governmental, societal and sometimes familial misunderstanding of their condition, it is essential that their medical care is provided in a safe, sustaining environment. This will support patients to participate fully in society, confident of what they need to stay as well as possible.

We have enough descriptions of the penalties of living with lupus. We have sufficient evidence of the undermining societal misunderstandings of the reality of fluctuation and fatigue. Action is now needed to build on and implement the growing body of evidence that non-pharmacological strategies, including better communication with patients [11], can improve medical outcomes for those with this debilitating, incurable, symptomatic illness. It is noticeable that survivors of COVID-19 are to be offered rehabilitation for their post-inflammatory fatigue and other symptoms to increase their chances of full recovery. Physicians, charities and patients need to be lobbying for comprehensive rehabilitation (cost-effectively with other disease groups) for people with SLE/UCTD [12].

Although ‘more research is needed’ on lupus-specific symptom-control strategies, improvements can be made immediately. Although a work in progress, the sustained effort to improve the communication skills of the multidisciplinary team in UK cancer units, facilitate access to supportive care and advocate for cancer patients’ needs has led to improved holistic care for people with cancer.

Early service experiences seem crucial. A multidisciplinary lupus ‘holistic needs assessment’ [13], preferably within 6 months of diagnosis or 3 months of serious relapse of lupus/UCTD of life-changing severity, could be evaluated for its effects on long-term morbidity, symptom severity and employment. This could include assessment and relevant treatment to reduce the impact of adverse childhood experiences, poverty and other disadvantage, manage symptoms and help patients to increase educational and employment opportunities, with referral to relevant charities and agencies. Earlier intervention for risk factors associated with longer term ill-health could increase the understanding of individual patients of how to mitigate their own health risks, introducing patients to self-management strategies before fatigue becomes established as an everyday experience, even in remission. A positive, proactive, rehabilitative approach might lead to better health outcomes in the longer term and lower societal costs. Establishment of peer-support networks seems to be cost effective and clinically effective in high-risk groups [14].

In the same way that academic, learned societies and lupus charities and journals publish position statements and guidelines on best practice on pharmaceutical management, these are now needed to highlight the symptoms and psychosocial impact of SLE. These could be used by patients in their contacts with employers, occupational health and benefits agencies. Efforts to involve patients in the development of guidelines and co-designing interventions could become the norm [15]. Excellence criteria for evaluating lupus units could require evidence that the departmental ethos includes: (a) patient-centred communication skills training; (b) engagement with the psychosocial consequences of lupus using both testimonies from individual patients and broader evidence from social science; (c) symptom control education; (d) availability of team support for patients in crisis; and (e) a drive to reach the most disadvantaged patients to offer maximal health support. Clinicians need to engage with social scientists in order that the valuable insights of these disciplines are not relegated to being an academic currency for career progression and make their deserved impact on clinical care and the lives of patients. The findings of social science research need to be accessible to patients.

Academic conferences, journals, research and grant-making programmes need to include symptom control and psychosocial issues (such as changes to employment practice to retain people with SLE in work) as core topics and highlight best practice in symptom control. If we do not act now, the changes so eloquently called for by patients in the study by Sloan *et al.* [3] will never be realized, and we will continue to read about, or live with, the debilitating consequences of this incurable disease, including significant personal and societal costs and time wasted in futile medical encounters.

## Data Availability

Data are available upon reasonable request by any qualified researchers who engage in rigorous, independent scientific research, and will be provided following review and approval of a research proposal and Statistical Analysis Plan (SAP) and execution of a Data Sharing Agreement (DSA). All data relevant to the study are included in the article.
